# Can p53, Ki-67 and bcl-2 predict biochemical failure after radical prostatectomy?

**DOI:** 10.4103/0970-1591.65390

**Published:** 2010

**Authors:** B. Baseskioglu, B. Akdogan, D. E. Baydar, H. Ozen

**Affiliations:** Hacettepe University Medical Faculty Urology Department, Ankara, Turkey; 1 Hacettepe University Medical Faculty Pathology Department, Ankara, Turkey

**Keywords:** Bcl-2, ki-67, microarray, p53, prostate cancer

## Abstract

**Background and Objective:**

To analyze p53, Ki-67 and bcl-2 expressions immunohistochemically and their predictive role in biochemical recurrence after radical prostatectomy.

**Materials and Methods:**

Seventy one patients who had undergone radical prostatectomy between 1992 and 2001 were randomly selected. Tissue microarrays were constructed from their radical prostatectomy specimens. They contained four cores from neoplastic and additional four cores from corresponding non-neoplastic regions. Gleason score ranged from 6-9, and pathological stage ranged from T2N0Mx to T3BN1. Staining for bcl-2 was scored visually taking percent negative, weak, moderate and strong positivity into consideration. Strong immunoreactivity was considered positive for p53. Ki-67 index was measured as the percentage of positive nuclei among tumor cells. Statistical analysis was performed to explore correlations between staining patterns and clinicopathological prognostic parameters.

**Results:**

The follow-up period extended from 13 to 112 months with a mean 60 (48 ± 23, 2) months. Of all, 38.02% had no evidence of disease, 52.1% were alive with disease and 9.8% were died during follow-up. The expression of p53, Ki-67 and bcl-2 in tumors were 39%, 76% and 5% respectively. While the secretory layer showed negative or weak bcl-2 staining in most cases, expression in basal cells was often stronger. Statistical analysis revealed differences in staining between normal and carcinoma for all three markers. There was no correlation between staining patterns and time to biochemical relapse. On the other hand, cases with higher Gleason sum showed the tendency for over expression of p53, Ki-67 and bcl-2 although the differences were not statistically different. Multivariate analysis revealed CMS group and seminal vesicle invasion as the independent predictors of PSA failure (log rank *P* = 0.0039 and *P* = 0.001, respectively).

**Conclusion:**

The proteins bcl-2, p53 and Ki-67 were expressed at a different rate in normal and neoplastic prostate tissue. Bcl-2 was mainly expressed by basal cells in normal glands. p53 and Ki-67 expression were increased in most prostate carcinomas. However, overall expression levels did not correlate with biochemical recurrence in this study.

## INTRODUCTION

Prostate cancer is the most common malignancy throughout the world among the males over middle age and the second leading cause of cancer-related deaths in western countries. Radical prostatectomy (RP) is the most preferred treatment option for patients with localized prostate cancer. Widespread usages of nerve-preserving technique resulted in a decrease in post-operative complications and increase in quality of life concerning predominantly continence and erectile functions, which made the technique more popular. However, 30-56% of the patients will recur in the first decade following surgery and even some will develop metastatic disease.[1] Many studies focused on to find accurate predictors of recurrence after RP over the past decade. The role of PSA and histopathological parameters in prognostic stratification of patients is still limited. New molecular-genetic researches are expected to bring new prognosticators to better define patients who will have more benefit from the surgery or who are in high risk group for the recurrence and could be better if treated with other modalities.

Certain studies focused on P53, Ki-67 and bcl-2 expressions to clarify the unknowns in biology and molecular genetics of prostate cancer. Conflicting evidence exists regarding prognostic influence of those markers. Some showed that they were significant prognostic parameters determining the recurrences,[2] whereas others have reported insignificant correlations.[3] Today, there is no unfailing indicator that can be used to foretell the disease prognosis in daily uro-oncology clinical practice.

In this study, the value of these three cell cycle proteins to predict recurrence was assessed together with other clinical and the histopathological parameters in intermediate and high risk prostatic carcinoma patients treated by radical prostatectomy at a single tertiary care center.

## MATERIALS AND METHODS

A total of 71 randomly selected prostate carcinoma patients who had been defined in intermediate to high risk group according to D’amico *et al*. criteria[4] and undergone radical prostatectomy (RP) between 1992 and 2001 were included in this study. Surgery was performed with retropubic approach, under the supervision of the same surgeon (HO). None of the patients received pre-operative hormonal treatment or radiotherapy. Sixty nine cases had pelvic lymphadenectomy. Pre-operative data including demographic, clinical and laboratory records were collected on all patients from the hospital charts.

Histopathological features of all cases were re-evaluated by the single pathologist (DEB). Tumor volume (estimated by simple eye-balling), Gleason score, localization, status of uni- versus bilaterality, focus number, surgical margins, presence of extra-prostatic extension, lymphovascular invasion, seminal vesicle invasion, lymph node metastasis and pathological stage (according to 2002 TNM classification) were noted.

### Tissue microarray (TMA) construction

Tissue microarrays were constructed by 0.6 mm tissue cores from 71 radical prostatectomies using a manual tissue arrayer (Beecher Instruments, Silver Spring, MD). They contained four cores from the index tumor (defined as most significant cancer nodule which was the largest tumor focus with the highest Gleason score and stage) and additional four cores from corresponding non-neoplastic regions for each case. Control tissues were placed in an orderly pattern throughout each array. Four-µm sections from the array blocks were cut for H-E and immunostaining. H-E sections were scanned to record the tissue type (non-neoplastic normal glands, prostatic intraepithelial neoplasia and adenocarcinoma) represented in each core.

### Immunohistochemistry

A total of 4-µm thick tissue sections taken from TMAs on charged slides were deparaffinized and rehydrated. Slides for bcl-2 were heated in citrate buffer steam (0.01 M, pH 8.0) for 20 min; the ones for p53 and Ki-67 were exposed to high temperature target retrieval in Dako target retrieval solution (cat#S1700) for 40 minutes to accomplish antigen unmasking. Endogenous peroxidase activity in sections was inactivated by Dako Endogenous Enzyme Block (S2001) for 5 min. Then the sections underwent incubation with primary antibodies. Anti-p53 antibody (Dako cat# M7001) was used at a dilution of 1:800 in antibody diluents buffer (ChemMate ADB250) for 45 minutes. Anti-Ki-67 antibody (Zymed cat# 18-0192) was applied at a dilution of 1:400 with overnight incubation at 4°C. Anti-bcl-2 antibody (Dako cat# M0887) was used at a dilution of 1:200 for 45 minute incubation. They were then washed in PBS (phosphate buffered saline). Specific immunoperoxidase stainings were developed using the DAKO Envision+ system (K 4007 horseradish peroxidase, Mouse Envision 3, 3’-diaminobenzidine Plus kit, DAKO) with 3, 3’-diaminobenzidine (DAB) as chromogen. DAB-stained slides were counterstained with hematoxylin, dehydrated, and mounted with a cover-slip.

### Quantization of immunohistochemistry

Immunostainings were evaluated microscopically for the represented tissue types (normal glands, prostatic intraepithelial neoplasia or carcinoma) in each core in the tissue microarrays. Intense nuclear staining involving more than 1% of the cells was considered positive for p53. Ki-67 labeling index (labeling frequency %) for benign and neoplastic areas was calculated by the following formula: [number of positive nuclei/total number of represented cells] ×100. <10% was scored as 1+ while ≥ 10% as 2+. Intensity and extent of cytoplasmic staining were recorded for bcl-2, visually taking per cent negative, weak, moderate and strong positivity into consideration. Moderate or intense level of staining involving more than 5% of the cells were considered positive, anything less than that was regarded as negative.

For the statistical analysis, Statistical Package for Social Sciences (SPSS) version 15.0 was used.

## RESULTS

Mean age and PSA values (±sd) at diagnosis were; 62.1 ± 6.5 years and 9.8 ± 6.5 ng/ml, respectively. Gleason score ranged from 6-9, and pathological stage ranged from T2N0 to T3BN1. Grade and stage characteristics of the cases were displayed in Table 1. Five patients had pelvic lymph node metastasis. Extraprostatic extension, seminal vesicle invasion, lymphovascular involvement and positive surgical margins were identified in 46 (64, 7%), 18 (25, 3%), 11 (15, 4%) and 31 (43, 7%) of the radical prostatectomies respectively.

**Table 1:** Distribution of cases in the arrays according to pT and Gleason score

Pathological Stage		Gleason score			Total	
		6	7	8 or >8
pT2	N0	12	8	2	22	23
	NX	1	0	0	1	
pT3a	N0	5	14	9	28	30
	N1	0	2	0	2	
pT3b	N0	0	11	3	14	18
	N1	0	2	1	3	
	NX	0	0	1	1	
Total		18	37	16	71	

Mean clinical follow-up for the patients was 60.48 (± 23.2). Biochemical relapse was observed in 36 (50.7%) patients. The mean time from surgery to recurrence was 36.4 months. Overall 5 year recurrence-free survival rate was 72.2%.

### TMAs and immunohistochemistry

Twenty seven cores contained only stromal tissue, but no prostate glands. Tissues from additional 19 cores were lost during the procedures for immunohistochemistry. Eventually no tumor tissue was available for immunohistochemical studies in two cases. One or more tissue cores containing non-neoplastic prostatic glands that could be evaluated were available for 66 different prostatectomies.

**Figure 1:**
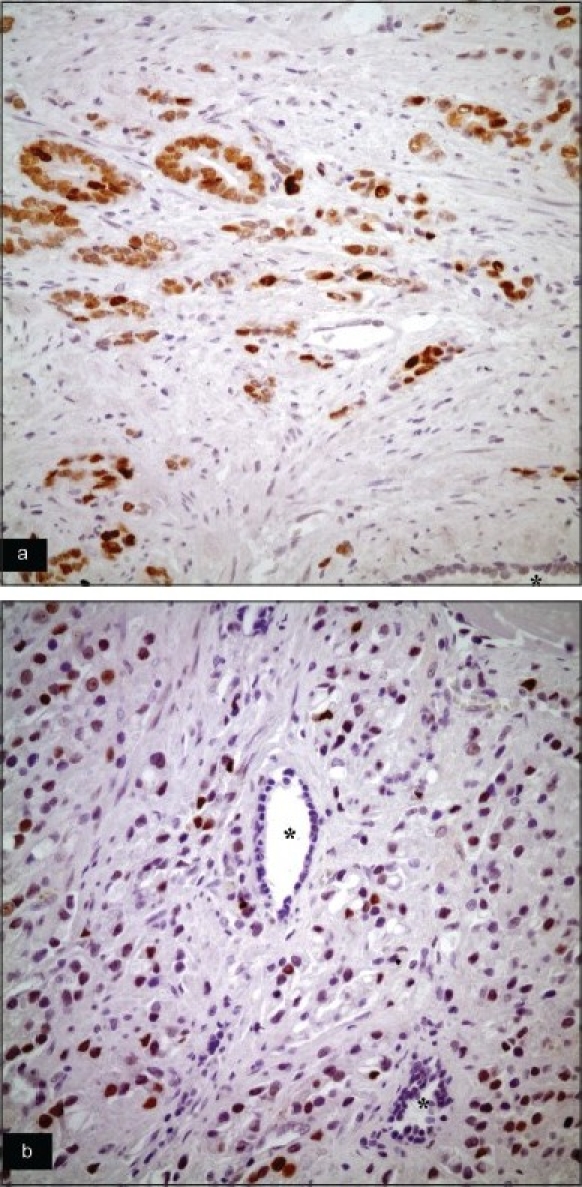
Two different cases of prostatic adenocarcinoma show diffuse p53 positivity. Non-neoplastic glands (indicated by ^*^) are negative (a and b: Anti-p53 primary Ab, ABC, ×400).

Nuclear p53 expression was found in more than 1% of cells in 29/69 tumors (42%). These tumors were considered p53 positive. Most of the time, positive p53 staining involved more than 50% of tumor cells [Figure 1]. Only four cases showed P53 over expression in non-neoplastic glands. In two of these, the glands were associated with adjacent chronic inflammation. The third had positive cells only at basal layer, not in secretory cells.

**Figure 2:**
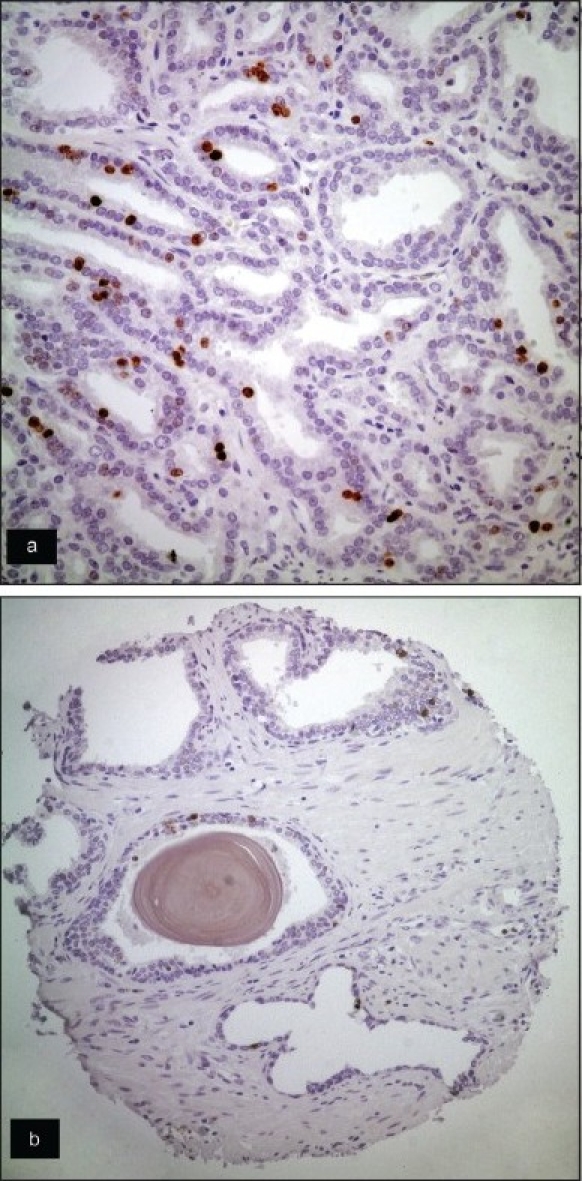
Ki67 labeling in a case of prostatic carcinoma (a) and its corresponding benign tissue (b) (A: Anti-Ki67 primary Ab, ABC, ×400; B: Anti-Ki67 primary Ab, ABC, ×200).

A high Ki-67 proliferation index (>10%) was found in 10% of 69 prostate cancer [Figure 2]. One case had Ki-67 positivity above 20%. None of the normal glands expressed Ki-67 over 10% although 1-10% positivity was detected in 40 cases among 69 where non-neoplastic glandular prostate tissue was available for evaluation.

**Figure 3:**
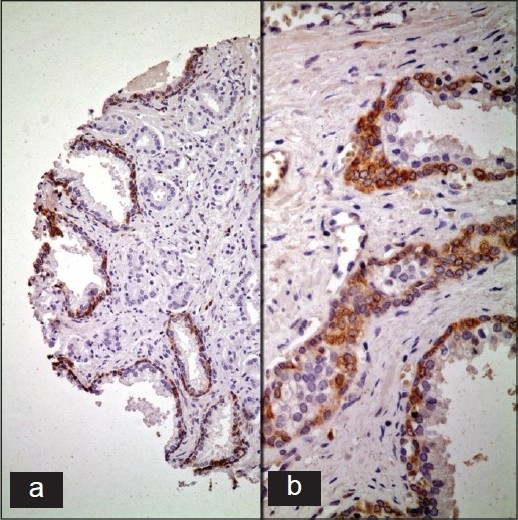
Shows bcl-2, which is selectively expressed by the basal cells of normal prostatic glands while the malignant acini are negative in most cases (a: Anti-bcl2 primary Ab, ABC, ×200; b: Anti-bcl2 primary Ab, ABC, ×400)

**Figure 4:**
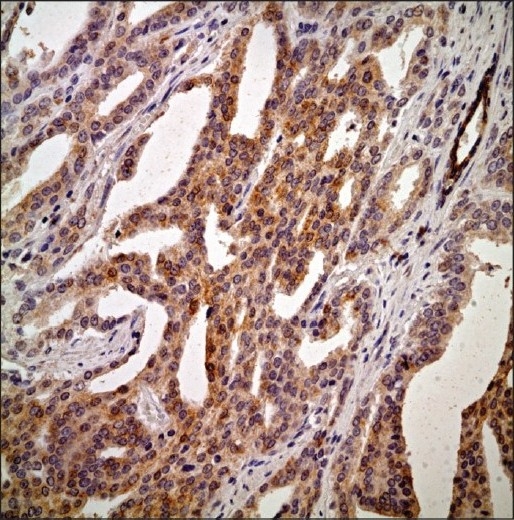
A rare case of prostatic adenocarcinoma demonstrating strong bcl-2 expression in this shown field (Anti-bcl2 primary Ab, ABC, ×400)

Bcl-2 showed diffuse cytoplasmic staining and typically restricted to the basal cell layer of normal glands [Figure 3]. 27 cases expressed bcl-2 in normal non-neoplastic acini (40.9%). In 10 of them basal cells were accompanied by positive secretory cells albeit in a lesser extent and intensity. Only four tumors (5.8%) revealed bcl-2 expression which was focally and involved less than 20% of neoplastic area [Figure 4].

There was no statistical correlation between p53, Ki-67 labeling and bcl-2 stainings.

None of the markers provided important prognostic information. There was no significant association of p53, Ki-67 or bcl-2 with pathologic stage, histological grade or the other prognostic variables as well as disease outcome in terms of biochemical failure or 5 year-disease free survival [Table 2]. In the univariate analyses, high Gleason score, bilateralism, presence of extraprostatic extension, seminal vesicle invasion, lymphovascular invasion, surgical margin positivity, pathologic stage, tumor volume over 5% and D’Amico high risk group were found significant for higher chance of biochemical failure [Table 3]. Gleason score, tumor focality (unifocal versus multifocal), status of seminal vesicle invasion and surgical margins, and CMS risk grouping were the determinants of five-year disease free survival [Table 4]. Multivariate analysis including these five parameters showed that only state of the seminal vesicle involvement and the CMS grouping proved to be of independent prognostic value, the relative risk of progression being 3.3 (*P* = 0.001) and 2.1 (*P* = 0.039), respectively [Table 5].

**Table 2:** Clinical and pathological variables in relation to immunohistochemistry*

	P53		Ki-67			Bcl-2	
	(-)	(+)	(-)	≤10%	>10%	(-)	(+)
Age							
≤ 62	20 (57, 1)	15 (42, 8)	7(20)	27 (77, 1)	1(2,8)	35 (100)	0(0)
>62	20 (58, 8)	14 (41, 1)	8 (23, 5)	20 (58, 8)	6 (17, 6)	30 (88, 2)	4(11,7)
PSA							
≤ 9,8	29 (59, 1)	20 (40, 8)	11 (22,4)	35 (71,4)	3 (6, 1)	46 (93, 8)	3 (6, 1)
> 9,8	11 (55)	9 (45)	4 (20)	12 (60)	4 (20)	19 (95)	1 (5)
Multifocality							
-	26 (60, 4)	17 (39, 5)	10 (23, 2)	30 (69,7)	3 (6,9)	42 (97, 6)	1 (2, 3)
+	14 (53, 8)	12 (46, 1)	5 (19, 2)	17 (65, 3)	4 (15,3)	23 (88,4)	3 (11, 5)
Bilaterality							
-	15 (65, 2)	8 (34,7)	4 (17, 3)	18 (78, 2)	1 (4, 3)	22 (95, 6)	1 (4, 3)
+	25 (54, 3)	21 (45, 6)	11 (23, 9)	29 (63, 04)	6 (13, 04)	43 (93, 4)	3 (6,3)
Gleason score							
<7	12 (66.)	6 (33, 3)	3 (16,6)	14 (77, 7)	1 (5, 5)	17 (94 4)	1 (5, 5)
≥7	28 (54, 9)	23 (45,9)	12 (23, 5)	33 (64,7)	6 (11,7)	48 (94, 1)	3 (5,8)
Epe							
-	18 (72)	7 (28)	8 (32)	14 (56)	3 (12)	26 (100)	0 (0)
+	22 (50)	22 (50)	7 (15,9)	33 (75)	4 (9,09)	39 (90, 6)	4 (9, 3)
Volume							
<%5	21 (60)	14 (40)	5 (14,2)	25 (71,4)	5 (14, 2)	35 (100)	0 (0)
≥%5	19 (55, 8)	15 (44, 1)	10 (29,4)	22 (64,7)	2 (5,8)	30 (88, 2)	4 (11,7)
SM							
-	24 (61 5)	15 (38, 4)	9 (23, 07)	28 (71,7)	2 (5, 1)	36 (92,3)	3 (7, 6)
+	16 (53, 3)	14 (46, 6)	6 (20)	19 (63, 3)	5 (16, 6)	29(96, 6)	1 (3, 3)
SVI							
-	31 (59, 6)	21 (40, 3)	11 (21, 1)	38 (73, 07)	3 (5,7)	49 (94, 2)	3 (5,7)
+	9 (52,9)	8 (47, 05)	4 (23, 5)	9 (52,9)	4 (23, 5)	16 (94, 1)	1 (5, 8)
LVI							
-	34 (58, 6)	24 (41,3)	14 (24, 1)	42 (72,4)	2 (3,4)	56 (96, 5)	2 (3, 4)
+	6 (54, 5)	5 (45, 5)	1 (9, 09)	5 (45, 4)	5 (45, 4)	9 (81, 8)	2 (18, 1)
LN metastasis							
-	37 (57, 8)	27 (42, 1)	14 (21, 8)	44 (68,7)	6 (9, 3)	60 (93,7)	4 (6, 2)
+	3 (60)	2 (40)	1 (20)	3 (60)	1 (20)	5 (100)	0 (0)
Pathologic stage							
pT2	15 (65, 2)	8 (34,7)	4 (17,3)	17 (73, 9)	2 (8,6)	21 (91, 3)	2 (8, 6)
pT3	25 (54, 3)	21 (45, 6)	11 (23, 9)	30 (65, 2)	5 (10,8)	44 (95, 6)	2 (4,3)
CMS groups							
Intermediate	27 (52, 9)	24 (47,05)	14 (27,4)	33 (64,7)	4 (7, 8)	49 (96, 07)	2 (3, 9)
High	13 (72, 2)	5 (27,7)	1 (5, 5)	14 (77 7)	3 (16, 6)	16 (88, 8)	2 (11, 1)
B. recurrence							
-	19 (54, 2)	16 (45, 7)	8 (22, 8)	24 (68, 5)	3 (8, 5)	33 (94, 2)	2 (5,7)
+	21 (61,7)	13 (38, 2)	7 (20, 5)	23 (67, 6)	4 (11,7)	32 (94, 1)	2 (5, 8)

* *P* values were above 0.05 for each crossing, Figures in parentheses are in percentage.

**Table 3:** Statistically important clinical and pathological variables in relation to biochemical recurrence

	Biochemical relapse(-) n=35	Biochemical relapse(+) n=36	P^*^
Bilaterality			
-	19	5	<0,01
+	16	31	
Gleason score			
<7	30	13	<0,01
≥ 7	5	23	
Extraprostatic ext.			
-	19	6	0,01
+	16	30	
Volume			
<%5	24	11	0,01
≥%5	11	25	
Surg, margin			
-	27	13	<0,01
+	8	23	SVI
-	32	21	<0,01
+	3	15	
SVI			
‐	32	21	<0,01
+	2	9	
LVI			
-	33	27	0,025
+	2	9	
Pathologic stage			
pT2	17	6	0,01
>pT2	18	30	
CMS groups			
Intermediate	34	17	<0,01
High	1	19	

**Table 4:** Prognosticators of five-year recurrence-free survival in univariate analysis

Variable	RFS (%) + SD	Log rank *P*
Multifocality		
-	69,9 + 0,07	0,043
+	79,5 + 0,09	
Gleason score		
<7	75,7 + 0,008	0,05
≥7	66.9 + 0,009	
Surgical margin		
-	89,4 + 0,005	0,005
+	55,8 + 0,009	
SVI		
-	86,6 + 0,05	0,000
+	35,6 + 0,12	
CMS risk group		
Intermediate	74,8 + 0,07	0,003
High	60 + 0,11	

SD: standard deviation

**Table 5:** Five-year recurrence-free survival in multivariate analysis

Variable	Hazard ratio	% 95 confidence	*P*
Multifocality			
-	0,630	0.295- 1.345	0,232
+			
Gleason score			
<7	1.288	0.551-3.012	0,559
≥7			
Surgical margin			
-	1.595	0.750-3.392	0,225
+			
SVI			
-	3.340	1.607-6.945	0,001
+			
CMS risk groups			
Intermediate	2.136	1.039-4.393	0,039
High			

## DISCUSSION

There has been ongoing interest to molecular pathways leading to malignant transformation and progression in prostate cancer. P53, Ki-67 and bcl-2 are the most intensely investigated gene products in various tumors which are involved in the regulation of cell cycle and/or apoptosis. In this study, we have examined P53, Ki-67 and bcl-2 expression immunohistochemically in intermediate and high risk prostate cancer and aimed to investigate their relationship with prognosis.

The p53 is a tumor suppressor gene, takes an important a role in cell cycle control, DNA repair, and apoptosis;[5,6] p53 gene mutations commonly occur in a wide variety of human malignancies. The most frequent form of p53 mutations are of the missense type and can result in an increased half-life of the p53 protein, making it detectable by immunohistochemistry.[7] Several previous studies have shown that alterations of the p53 are less frequent in prostate cancer than in other common tumors.[8–10] The prevalence of p53-positivity in prostate tumors has been found in the range of 4% -60%.[11,12] Our study showing 42% p53 immune staining falls in between. We have also observed that normal prostate glands displayed positive reaction for p53 in four cases. However, in two of these, the glands were intimately associated with prominent inflammatory infiltrate, which can induce up-regulation of the protein understandably. In another case, p53 positive cells were noted interestingly in the basal layer. We could not determine a significant association between p53 overexpression and prognosis although RFS was found to be lower in those with (+) p53 expression, compared to those (-). Risk factors for recurrence such as high Gleason score, EPU, surgical margin (+) and involvement of the seminal vesicle were also more frequently detected in p53 (+) patients, but these didn’t reach statistical significance.

Bcl-2 is known to have strong inhibitory effect on apoptosis.[13] So it is likely that bcl-2 overexpression can facilitate prostate cancer progression through prolonged tumor cell survival and an increased net tumor.

Additionally, cells that are unable to undergo apoptosis can accumulate secondary genomic aberrations. Thus, the accumulation of additional genomic aberrations may be ultimately responsible for tumor progression and poor prognosis of Bcl-2-positive prostate carcinomas. There are studies, claiming that increase in bcl-2 expression can be an important marker for PSA recurrence following radical prostatectomy.[9] Previous clinical studies have suggested a poor prognosis of hormonally treated Bcl-2-positive prostate carcinomas.[14,15] Increased bcl-2 expression during the early stage of prostate cancer is reported in 32-41%.[16] Interestingly, in our series, the rate of tumor bcl-2 staining is very low, being only 5.8%. Rather benign prostate glands are frequently stained with the strongest expression by the basal cells. We could not observe any correlation between bcl-2 expression in the tumor and prognostic variables or PSA recurrence. Our results are similar to those of Borre *et al*, who have found that positivity of bcl-2 is 10% in prostate cancer and it is not an independent prognostic factor.[17]

Several groups have reported that an increased tumor cell proliferation is of prognostic relevance in prostate cancer.[18,19] Ki-67 is a proliferative nuclear antigen. There are studies where Ki-67 labeling index (LI) appears as an independent prognostic factor prostate cancer.[20] Moul *et al*, have stated that, patients with a high MIB-1 score -a monoclonal antibody to recombinant parts of Ki-67- experienced recurrence 3.1 times more than others.[21] We have observed a high (over 10%) Ki-67 LI in 10% of our tumors. However, we could not see a prognostic impact of Ki-67 proliferative activity in our patient set.

The analysis of biochemical failure in our patient series has showed a strong association between Gleason score, bilateralism, extraprostatic extension, seminal vesicle invasion, lymphovascular invasion, surgical margin positivity, pathologic stage, tumor volume and CMS. This is consistent with previous reports.[22–24] Multiparameter analysis by the Cox proportional hazard model has showed that only Gleason score and a CMS are independent predictors of 5-year disease free survival.

In summary, overexpression of p53, bcl-2 and high Ki-67 proliferative index do occur in prostate cancer. They may be important in the carcinogenesis; however, we could not find them influential in the disease progression. Attempts should go on to discover better biomarkers to better predict failure after RP. We have to admit that, genetic biomarker studies on prostate cancer are still in their infancy stages.
